# Opinion and uptake of chloroquine for treatment of COVID-19 during the mandatory lockdown in the sub-Saharan African region

**DOI:** 10.4102/phcfm.v13i1.2795

**Published:** 2021-06-15

**Authors:** Uchechukwu L. Osuagwu, Obinna Nwaeze, Godwin Ovenseri-Ogbomo, Richard Oloruntoba, Bernadine Ekpenyong, Khathutshelo P. Mashige, Chikasirimobi Timothy, Tanko Ishaya, Raymond Langsi, Deborah Charwe, Emmanuel Kwasi Abu, Miner A. Chundung, Kingsley E. Agho

**Affiliations:** 1 School of Medicine, Diabetes, Obesity and Metabolism Translational Research Unit, Western Sydney University, Campbelltown, New South Wales, Australia; 2 African Eye and Public Health Research Initiative, African Vision Research Institute, Discipline of Optometry, University of KwaZulu-Natal, Durban, South Africa; 3 Department of Public Health, Faculty of Health Science, NHS, Leeds, United Kingdom; 4 Department of Optometry, College of Applied Medical Sciences, Qassim University, Saudi Arabia; 5 Department of Optometry and Vision Science, Faculty of Life Sciences, University of Benin, Benin City, Nigeria; 6 School of Management, Faculty of Health Sciences, Curtin Business School, Bentley, Western Australia, Australia; 7 Department of Public Health, Faculty of Allied Medical Sciences, College of Medical Sciences, University of Calabar, Calabar, Cross River State, Nigeria; 8 African Eye and Public Health Research Initiative, African Vision Research Institute, University of KwaZulu-Natal, Durban, South Africa; 9 Department of Optometry and Vision Science, Faculty of Health Sciences, Masinde Muliro University of Science and Technology, Kakamega, Kenya; 10 Department of Computer Science, Faculty of Health Sciences, University of Jos, Jos, Nigeria; 11 Health Division, Faculty of Health Sciences, University of Bamenda, Bambili, Cameroon; 12 Department of Food and Nutrition, Tanzania Food and Nutrition Centre, Dar es Salaam, Tanzania; 13 Department of Optometry and Vision Science, School of Allied Health Sciences, University of Cape Coast, Cape Coast, Ghana; 14 Department of Community Medicine, College of Health Sciences, University of Jos, Jos, Nigeria; 15 School of Health Sciences, Western Sydney University, Campbelltown, New South Wales, Australia

**Keywords:** coronavirus, sub-Saharan Africa, chloroquine hydrochloride, Africa, poisoning

## Abstract

**Background:**

As the search for effective treatment of coronavirus disease 2019 (COVID-19) infection continues, the public opinion around the potential use of chloroquine (CQ) in treating COVID-19 remains mixed.

**Aim:**

To examine opinion and uptake of CQ for treating COVID-19 in the sub-Saharan African (SSA) region.

**Setting:**

This study was conducted through an online survey software titled SurveyMonkey.

**Methods:**

Anonymous online survey of 1829 SSA countries was conducted during the lockdown period using Facebook, WhatsApp and authors’ networks. Opinion and uptake of CQ for COVID-19 treatment were assessed using multivariate analyses.

**Results:**

About 14% of respondents believed that CQ could treat COVID-19 and of which, 3.2% took CQ for COVID-19 treatment. Multivariate analyses revealed that respondents from Central (adjusted odds ratios [aOR]: 2.54, 95% confidence interval [CI] 1.43, 4.43) and West Africa (aOR: 1.79, 95% CI 1.15, 2.88) had higher odds of believing that CQ could treat COVID-19. Respondents from East Africa reported higher odds for uptake of CQ for COVID-19 than Central, Western and Southern Africans. Knowledge of the disease and compliance with the public health advice were associated with both belief and uptake of CQ for COVID-19 treatment.

**Conclusion:**

Central and West African respondents were more likely to believe in CQ as a treatment for COVID-19 whilst the uptake of the medication during the pandemic was higher amongst East Africans. Future intervention discouraging the unsupervised use of CQ should target respondents from Central, West and East African regions.

## Introduction

Global public health authorities must combat dangerous and unproven theories about the use of the antimalarial, chloroquine (CQ), for treating coronavirus disease 2019 (COVID-19) infections despite lack of evidence. Since the declaration of the COVID-19 pandemic by the World Health Organization (WHO) on 11 March 2020,^1^ vaccines are now being introduced in different countries for the control of the infection^2^ but their effectivity is still to be tested.^3^ Although novel treatments and/or vaccines will take time to be distributed amongst patients, there is growing interest in the use of existing medications, such as CQ and hydroxychloroquine (HCQ), as potential treatments of COVID-19.^4,5,6,7^ Despite promising *in vitro* results,^8^ there are no direct supporting data on the effective role of CQ and HCQ in the treatment of COVID-19.^9^ Those reporting that the drug has a favourable effect on the outcomes of COVID-19 were not clinical trials and used poor methodology.^4,5,6,7,10^

Chloroquine and its analogue, HCQ are considered safe and have side effects that are generally mild and transitory. However, there is a narrow margin between the therapeutic and toxic dose, and CQ poisoning has been associated with life-threatening cardiovascular disorders and^11^ irreversible blindness from CQ retinopathy.^12^ Also, treatment with HCQ has been associated with in-hospital mortality in patients with COVID-19 in New York State.^1^ Chloroquine is proven effective as an antimalarial, amoebicide and antirheumatic, and its possible adverse reactions are well documented.^13^ The use of this medication outside of these conditions should be appropriately monitored in the hospital as required by the Emergency Usage Authorisation (EUA) or in a clinical trial with appropriate screening and monitoring.^14,15^

Early on in the pandemic, the media environment was awash with misinformation concerning the use of CQ in the treatment of the COVID-19 infection. Layered on top of this was the retraction on 04 June 2020 of the *Lancet* paper, which claimed that treating COVID-19 with the antimalarial drug raised the heart-related death risk for COVID-19 patients in the hospital without showing any benefit.^16^ The study was the basis for the halt of many studies of the antimalarial by the WHO. The indiscriminate promotion of this medication by those in authority and widespread use of CQ in Africa have led to extensive shortages, self-treatment and fatal overdoses.^1^ The shortages and increased market prices of this medication left the already weak health systems in Africa vulnerable to substandard and falsified medical products.^15^ Governments in sub-Saharan African (SSA) countries are ‘strongly considering’ putting prescription monitoring programs in place to ensure that off-label use of CQ and HCQ is appropriate and beneficial for COVID-19 patients.^15^

Considering the public-health emergency nature of COVID-19 and the new challenges of the second wave in SSA countries,^17^ it is necessary to investigate the perception and behaviour of Africans regarding CQ use for COVID-19. This study sought the opinions of people from SSA countries about the belief that CQ can cure COVID-19, and the influence of such a belief on their behaviour by purchasing the medication to treat the infection and the factors associated with these variables. This study assessed the relationship between respondents’ belief and use of CQ as a cure for COVID-19 and the compliance to the mitigation practices put in place by the respective governments to limit the spread of the virus. The findings are important for planning strategies for the control of COVID-19 and future outbreaks, and will help to identify the population at greater risk of CQ abuse, which can be targeted to prevent complications as the pandemic still unfolds. Also, the findings will help to design interventions that will minimise the indiscriminate and/or unauthorised use of this medication amongst the population.

## Methods

### Study design

This self-administered web-based survey was conducted during the mandatory lockdown period (27 April 2020 – 17 May 2020) in most of the countries surveyed. It was not feasible to perform a nationwide community-based sample survey during the lockdown period, so data were obtained electronically through SurveyMonkey. The questionnaire included a brief overview of the context, purpose, procedures, nature of participation, privacy and confidentiality statements and notes to be filled. Informed consent and permission to use de-identifiable information in the publication were obtained from the respondents. Information was sought on the respondents’ knowledge of the causes and symptoms of COVID-19 using the WHO validated tool.^18^ Respondents were also asked about their belief on the use of CQ for the treatment of COVID-19, and if they had purchased and used CQ during the COVID-19 pandemic to avoid contracting the virus.

Prior to the launching of the survey, a pilot study was conducted to ensure clarity and understanding as well as to determine the duration for completing the questionnaire. Participants (*n* = 10) who took part in the pilot study were not part of the research team and did not participate in the final survey as well. This self-administered online questionnaire consisted of 58 items divided into four sections (demographic characteristics, knowledge, attitude, perception and practice).

### Setting

The questionnaire was disseminated on social media platforms (Facebook and WhatsApp) commonly used by the locals in the participating countries. Emails sent to authors’ contacts and contact groups were also used by the researchers to facilitate response. On all platforms, recipients were encouraged to share the e-link of the survey with others.

### Study population and sample size determination

Data were collected from four SSA regions including Western, Eastern, Southern and Central Africa which consisted of people from Ghana, Cameroon (English speaking populations), Nigeria, South Africa, Tanzania, Kenya, Uganda, Malawi, Rwanda, etc. Classification of countries into regions was based on the regions of the African Union.^19^ To be eligible for participation, participants had to be 18 years and over, able to read and understand English and should be able to provide online consent.

The study assumed a proportion of 50% because the main objective of this research was on COVID-19 and no previous study from the SSA region has examined factors associated with belief and uptake of CQ as a cure for COVID-19 during the pandemic. For expected proportion with 2.5% absolute precision and 90% confidence, an online sample size calculator^20^ determined that a sample size of approximately 1408 including 30% non-response rate was required to detect significant differences because it was an online survey. The sample size of 1829 participants used in this study is large enough to detect any statistical differences.

### Independent variables

The independent variables included demographic (age, gender, marital status, country of origin [with Southern Africa as the base], education, employment and religion), practice (included compliance to mitigation practices of handwashing, self-isolation, quarantine and use of facemask when going out) and risk perception. Variables were summarised as counts and percentages for categorical variables.

### Dependent variables

The dependent variables were the belief on the effectiveness of CQ for COVID-19 treatment, and purchase of the medication for COVID-19. Participants were asked the following questions: ‘Do you believe that COVID-19 can be cured by taking CQ tablets?’ and ‘have you purchased CQ for COVID-19?’. Responses were categorised as ‘Yes’ (1) or ‘No’ (0).

### Data analysis

All analyses were performed in Stata version 14.1 (Stata Corp 2015, College Station, Texas, United States [US]). A two-way frequency table was used to obtain the prevalence estimates of those who believed that CQ could be used to treat COVID-19 and those who purchased the CQ. In the univariate analyses, odds ratios with 95% confidence intervals (CI) were calculated in order to assess the unadjusted risk of the independent variables on selected covariates. Multiple logistic regression analyses used pooled data of the four sub-regions and different key dependent variables to examine their relationship with the number of years of formal education of the respondents. Also, the logistic regression was used to determine whether any observed effect persisted in the presence of possible confounding variables. In addition, the study determined whether the acquisition of CQ was influenced by the respondent’s knowledge and compliance with mitigation practices put in place to stop the spread of the infection. Details of the questions were utilised to derive scores for knowledge; compliance with mitigation practices was presented in the Supplementary Table 1.

**TABLE 1:** Descriptive statistics for socio-demographic characteristics, knowledge, risk perception and compliance to practices towards the coronavirus disease 2019 infection.

Variables	*n*	%
**Age category, in years (*n* = 1800)**		
18–28	685	38.06
29–38	488	27.00
39–48	401	22.28
49+	226	12.56
**Sex (*n* = 1801)**		
Males	1005	55.8
Females	796	44.00
**Sub-region (*n* = 1773)**		
West Africa	999	56.4
East Africa	185	10.4
Central Africa	220	12.4
Southern Africa	369	20.8
**Employment status (*n* = 1809)**		
Employed	1205	67.00
Unemployed	604	33.39
**Marital status (*n* = 1805)**		
Married	802	44.43
Not married	1003	56.00
**Religion (*n* = 1806)**		
Christianity	1596	88.37
Others	210	11.63
**Highest level of education (*n* = 1809)**		
Postgraduate degree (Masters/PhD)	600	33.17
Bachelor’s degree	986	54.51
Secondary/Primary	223	12.33
**Profession**		
Non-healthcare sector	1324	77.16
Healthcare sector	392	22.84
**Do you live alone during COVID-19 (*n* = 1807)**		
No	1474	81.57
Yes	333	18.43
**Compliance**		
**Practised self-isolation (*n* = 1792)**		
No	1231	68.69
Yes	561	31.31
**Home quarantined because of COVID-19 (*n* = 1789)**		
No	1084	60.59
Yes	705	39.41
**Worried about contracting the infection (*n* = 1829)**		
Very worried	574	31.38
Worried	675	36.91
Not worried	580	31.71
**Knowledge of COVID-19 transmission**†		
Inadequate (0–2 points)	1334	72.94
Adequate (3–4 points)	495	27.06
**Knowledge of symptoms**‡		
Inadequate (0–6 points)	1180	64.52
Adequate (7–9 points)	649	35.48
**Perception of risk of contracting the infection**§		
Inadequate	958	52.38
Adequate	871	47.62
**Compliance to mitigation practices**		
Low	484	26.46
Moderate	1057	57.79
High	288	15.75

*N = 1829*

COVID-19, coronavirus disease 2019; PhD, Doctor of Philosophy.

† , the maximum score was 4 points;

‡ , maximum score was 9 points;

§ , maximum score was 24 points. Mitigation practices included those put in place by the African governments and included hand hygiene, use of facemasks, social distancing during the lockdown, not attending large gatherings including religious events.

### Ethical considerations

Ethical approval for the study was sought and obtained from the Human Research Ethics Committee of the Cross River State Ministry of Health (CRSMOH /HRP/HREC/2020/117). The study was carried out in accordance with the Helsinki Declaration for Human Research. The confidentiality of participants was assured in that no identifying information was obtained from participants. The study adhered to the tenets of Helsinki’s declaration, and informed consent was obtained from all participants prior to completing the survey. Participants were required to answer a ‘yes’ or ‘no’ to the consent question during survey completion to indicate their willingness to participate in this study.

## Results

### Characteristic of the sample

**FIGURE 1:**
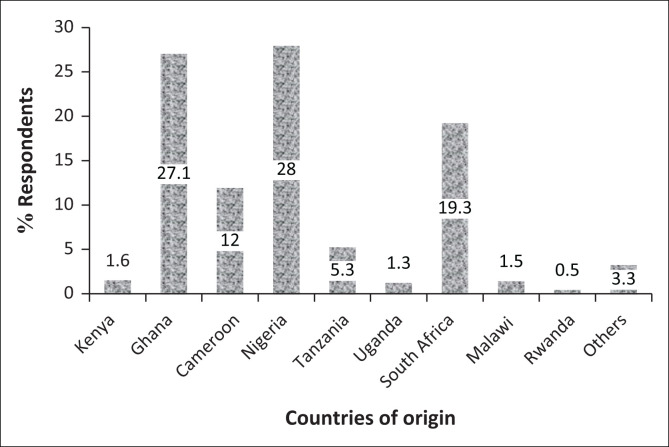
Percentage distribution of the respondents by country of origin (*n* = 1829) in sub-Saharan Africa.

A total of 1829 adults responded to the outcome of interest in the survey and consisted of respondents from four SSA regions. The distribution of respondents by country of origin is shown (Figure 1). The mean age was 26 years (range 18–50 years); many were aged 18–28 years (38.1%). More than half of the respondents were from Western Africa with a majority (91.3%) of the residents in their home country at the time of this study. Up to 87.7% had a university degree or higher education (Table 1). The majority were non-healthcare workers and did not live alone at the time of the COVID-19 lockdown.

Most (68.7%) of the African respondents practised self-isolation during the pandemic, whilst 60.6% of them were quarantined at the recommendation of health officers. Many respondents expressed some worry about contracting the virus and knowledge of the transmission and symptoms of the infection were generally inadequate amongst the respondents, as shown (Table 1).

### Prevalence of the belief and uptake of chloroquine for the coronavirus disease 2019 treatment during the pandemic

**FIGURE 2:**
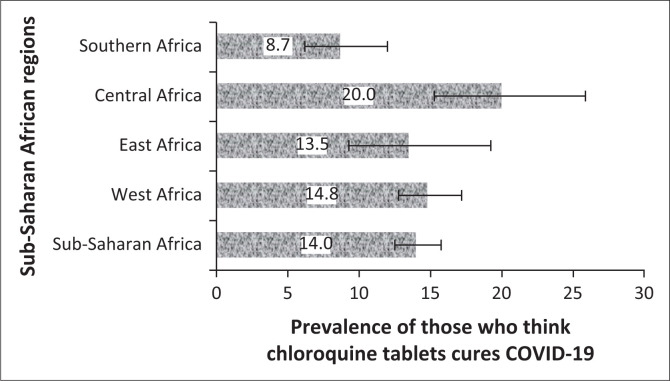
Prevalence and 95% confidence intervals of the belief in chloroquine tablets for the coronavirus disease 2019 treatment in sub-Saharan African regions.

**FIGURE 3:**
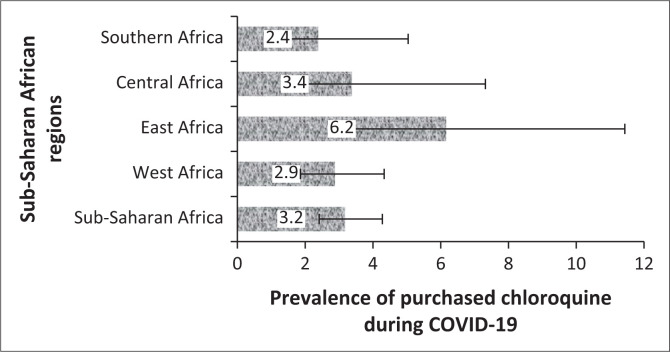
Prevalence and 95% confidence intervals of chloroquine use for the coronavirus disease 2019 treatment in sub-Saharan African regions.

The prevalence and 95% CI of the belief in CQ as a cure for COVID-19 and uptake during the COVID-19 pandemic for the four sub-regions, respectively, are shown (Figure 2 and Figure 3). The prevalence of belief in CQ as a cure for COVID-19 was significantly higher in Central Africa (20, 95% CI: 15.2–25.8) and lower in Southern Africa (9, 95% CI: 6.2– 12.0; *p* = 0.001). Although there was higher uptake of CQ amongst East Africans during the pandemic, the difference was not statistically significant (*p* = 0.174). Of the 47 respondents in SSA regions who purchased CQ for COVID-19, 19 of them (40.4%) did not believe that CQ was an effective treatment for COVID-19.

### Univariate analysis

The unadjusted odds ratios and 95% CI of perceived effectivity of CQ and uptake amongst respondents in this study are presented (Table 2). From the table, respondents living in Central Africa (unadjusted odds ratio, OR: 2.63, 95% CI: 1.61–4.30) and West Africa (OR: 1.83, 95% CI: 1.22–2.74) were more likely to believe that CQ can cure COVID-19; however, age and educational status were not associated with any of the outcome variables in this cohort. By contrast, no significant association was observed between the uptake of CQ for the COVID-19 treatment and any of the demographic variables. Belief in the use of CQ and its uptake during the pandemic were not dependent on whether the participants lived in their country of origin or outside their country of origin.

**TABLE 2:** Prevalence and unadjusted odds ratios (95% confidence intervals) for factors associated with belief and uptake of chloroquine tablets for treating the coronavirus disease 2019 and uptake in response to the coronavirus disease 2019 outbreak in four sub-Saharan African regions.

Variables	Perception			Uptake		
	Prevalence	OR	95% CI	Prevalence	OR	95% CI
**Sub-region**						
Southern Africa	8.67	1.00	-	2.43	1.00	-
Central Africa	20.00	2.63	1.61–4.30	3.37	1.40	0.46–4.24
East Africa	13.51	1.65	0.94–2.87	6.16	2.64	0.96–7.23
West Africa	14.81	1.83	1.22–2.74	2.88	1.19	0.50–2.81
**Knowledge of COVID-19**						
**Transmission**†						
Inadequate	8.92	1.00	-	2.99	1.00	-
Adequate	28.69	4.11	3.13–5.39	4.08	1.38	0.75–2.52
**Symptoms**‡						
Inadequate	14.92	1.00	-	3.41	1.00	-
Adequate	13.10	0.86	0.65–1.00	3.13	0.91	0.50–1.69
**Perception of risk of contracting the infection**§						
Low risk (0–13)	15.66	1.00	-	3.00	1.00	-
High risk (14–24)	12.74	0.79	0.60–1.03	3.64	1.22	0.68–2.19
**Compliance to mitigation practices**	-	-	-	-	1.00	-
Low	13.0	1.00	-	1.41	1.00	-
Moderate	13.5	1.05	0.76–1.44	3.37	2.44	0.95–6.37
High	19.1	1.58	1.06–2.34	6.01	4.47	1.59–12.60

COVID-19, coronavirus disease 2019; OR, odds ratio; CI, confidence interval.

Only variables with significant association are shown. Confidence intervals (CIs) excluding ‘1’ are statistically significant at *p* < 0.05 level;

† , the maximum score was 4 points;

‡ , maximum score was 9 points;

§ , maximum was 24 points. Mitigation practices included those put in place by the African governments and included hand hygiene, use of facemasks, self-isolation, social distancing during the lockdown, not attending large gatherings including religious events.

Respondents who perceived CQ as a cure for COVID-19 were more likely to be those who demonstrated adequate knowledge of how the virus is transmitted (OR: 4.11, 95% CI: 3.13–5.39). They were also more likely to highly comply with the mitigation practices (OR: 1.58, 95% CI: 1.06–2.34) put in place by the respective African governments to stop the spread of the virus during the pandemic. High compliance with the mitigation practices increased the odds of the demonstrated practice of purchasing CQ for the treatment of COVID-19 by up to 4.5 folds compared to those who had poor compliance with the mitigation practices.

### Multivariate analysis

The multivariate analysis, which was adjusted for all potential cofounders, is presented (Table 3). It was revealed that belief in the use of CQ for COVID-19 was predominant amongst respondents living in Central and West Africa, and was associated with adequate knowledge of the disease transmission (adjusted odds ratio [aOR] 4.59, 95% CI: 3.38–6.23). By contrast, uptake of CQ during the pandemic was 3.18 folds (95% CI: 1.02–9.94) higher amongst East Africans than Southern Africans, after controlling for all the potential cofounders and was associated with high knowledge of the disease transmission and compliance with the mitigation practices during the outbreak.

**TABLE 3:** Adjusted odds ratio (95% confidence intervals) of belief and uptake of chloroquine tablets for treating the coronavirus disease 2019.

Variables	Perception		Uptake	
	aOR	95% CI	aOR	95% CI
**Sub-region**				
Southern Africa	1.00	-	1.00	-
Central Africa	2.54	1.43–4.43	1.69	0.49–5.92
East Africa	1.61	0.85–2.93	3.18	1.02–9.94
West Africa	1.79	1.15–2.88	1.48	0.54–4.06
**Knowledge of COVID-19**				
**Transmission**†				
Inadequate	1.00	-	1.00	-
Adequate	4.59	3.38–6.23	2.03	1.04–3.97
**Symptoms**‡				
Inadequate	1.00	-	1.00	-
Adequate	0.89	0.65–1.22	1.13	0.58–2.21
**Compliance to mitigation practices**	1.00	-	1.00	-
Low	1.13	0.77–1.65	2.23	0.75–6.62
High	1.56	0.96–2.55	4.33	1.30–14.40

COVID-19, coronavirus disease 2019; aOR, adjusted odds ratio; CI, confidence interval.

Only variables with significant association are shown. Confidence intervals excluding ‘1.00’ are statistically significant at *p* < 0.05 level;

† , the maximum score was 4 points;

‡ , maximum score was 9 points. Mitigation practices included those put in place by the African governments and included hand hygiene, use of facemasks, self-isolation, social distancing during the lockdown, not attending large gatherings including religious events.

## Discussion

This study provided the first comprehensive evidence on belief in the CQ controversy for COVID-19 treatment perception and behaviour amongst the African population. It provides important knowledge to manage the evolving COVID-19 pandemic in the region. One in seven respondents believed that CQ can cure COVID-19, particularly Central and West Africans and those with adequate knowledge of the disease transmission. East Africans, and those that complied with the government mitigation practices, were also more likely to purchase CQ for COVID-19. The behaviour to purchase CQ tablets for COVID-19 contradicts the WHO and the US Food and Drug Administration (FDA) warnings against the use of CQ for COVID-19.^14,15^

The belief that CQ could cure COVID-19 and therefore be used indiscriminately for the same may be impacting the lives of others who depend on CQ for the approved uses.^21^ As shown in this study, more than two-thirds of those who purchased CQ did not believe in its use for COVID-19 treatment, suggesting that they may have bought the medication just for stocking to avoid possible future market shortage of the drug, should it be proven that it was effective in treating COVID-19. Storage of the medication was already causing shortages across the region and had the potential to further increase the panic amongst those who depend on this medication for their medical conditions.^1^ The finding that people with adequate knowledge of the disease transmission were more likely to purchase CQ might be because of information overload and medication misinformation regarding cures for COVID-19 that have been shown to spread unnecessary fear and panic leading members of the public to undermine legitimate public health advice.^22^ Majority of the respondents were young people, were more likely to have internet access and were probably more exposed to the media, which may not necessarily translate into an increase in actual knowledge. Exposure to the media might enhance the impression of one’s knowledge or self-perceived knowledge, as reported previously.^23^ Identifying this group of people and discouraging them from indiscriminate use of CQ certainly become a responsible public health approach.

The belief and uptake of CQ amongst the respondents may have also been encouraged by the socio-behavioural factors of familiarity with the drug and its perceived efficacy.^24^ This may explain the lack of association between the outcome variables and educational level in this study. Interestingly, we also found that those who were highly compliant with the government regulations to stop the spread of the disease were also more likely to endorse the CQ misinformation. This finding contrasts with those who believe in conspiracy theories such as the origin of the disease and vaccine efficacy, who have been found to be less likely to be compliant to government regulations.^25,26,27^ The former is more likely driven by fear of contracting the disease whilst the latter is driven by mistrust.

The CQ controversy became the focus of global scientific, media and political attention after a French virologist went public on social media to promote the use of CQ to treat or prevent COVID-19.^28^ His opinion was widely picked up by people across the globe, and many demanded immediate CQ for all.^29^ Despite other studies that have shown that CQ may not be as efficacious as claimed especially in severe cases,^30,31,32^ it still resulted in a scarcity for those who were on CQ/HCQ for legitimate indications such as malaria and lupus. According to the WHO guidelines, CQ is restricted and strictly reserved for severe malaria and special cases of uncomplicated malaria in patients allergic to other drugs.^33,34^ Although CQ has been removed as a first line treatment regimen for malaria caused by *Plasmodium falciparum* in SSA countries,^35^ it is still available as an over-the-counter (OTC) medicine in many of them.^28,33^ The fear of contracting the disease as seen in 68% of the respondents who were ‘worried about contracting COVID-19’ may have driven people to buy whatever the media promoted as a cure for the disease. This behaviour has spread beyond CQ to include zinc supplements, aspirin, vitamin C and azithromycin.^36^

Generally considered safe for the well-known approved indications in Africa, intake of CQ has been associated with severe adverse effects in COVID-19 patients. Patients with underlying health issues, such as heart and kidney disease, are more likely to be at increased risk of experiencing heart problems when taking CQ and HCQ according to the FDA.^37^ This becomes more disturbing in Africa where many have underlying diseases they are unaware of because of poor health systems and or lack of proper screening programs. With this in mind, and in the light of recent evidence that CQ and HCQ are not effective for the treatment of COVID-19,^9^ this study will guide SSA countries in formulating temporary prescription guidelines and restrictions around CQ usage. One way of doing this is through legislation of CQ/HCQ as prescription-only-medication and making it available to designated pharmacies within regions. In effect, with CQ/HCQ as prescription-only-medicine, physicians would be ‘forced’ professionally to state the actual indication for any prescriptions given. The current frontline drugs for malaria are the artemisinin-based combination therapies (ACTs) which are also OTC prescriptions.^38^ These medications can be subsidised for this period by governments to make them accessible to the populace.^39^ This study also recommends that physicians should place some emphasis on medication history of their patients to identify those who do not need the medication but are taking it, as well as using such encounters to counsel patients on medication safety and associated adverse effects. More importantly, the present finding would encourage concerted health promotional activities through campaigns at various governmental levels on educating the people on the dangers of self-medication through radio and television as well as via the commonly used social media platforms in each country. The media strategy was effective during the swine flu outbreak.^40^ A series of public service announcements can be crafted, and made available in both English and French to increase awareness of COVID-19. Such announcements should encourage testing and medical checks for symptomatic patients, through emphasis on the benefits of testing, overcoming drug misinformation and increasing people’s perceptions of their own ability to control the spread of the disease.

Formal education most often teaches basic reading skills, enlightens and aids in removing some of the cultural ideologies that lead to the misconceptions that affect proper and adequate prevention and treatment of diseases. Although studies in Africa have shown a significant association between higher levels of education and positive knowledge, attitude and practice towards diseases like malaria,^41^ as well as with recognition and appropriate treatment of diseases,^42,43^ we found no association between level of education and both perception and uptake of CQ for COVID-19. This was despite the fact that in this study, there was a preponderance of highly educated people, although not reflective of the general population of the region.

### Strengths and limitations

Firstly, the survey was only administered online. It may not have captured the opinion of those in rural areas where internet penetration remains relatively low^44^ and older people who are less likely to use internet compared to younger ones. As the increase in public interest during the pandemic resulted in greater internet use,^45^ this may not have a great impact on the findings coupled with the fact that it was the only reliable means to disseminate information at the time of this study. This was also an innovative way to provide real-time data on the current situation. Secondly, the survey was available only in English, making it impossible for some SSA francophone countries to participate, and the result may not be generalisable to all sub-Saharan African population because of the sampling technique. Thirdly, there were wide variations in the response rate per region, which may be because of population differences and poverty levels that influence access to internet. Fourthly, the lack of incentive and not receiving assistance with any online company for distribution of the survey may have affected the reach of the survey. It also meant that the social media accounts could not be verified and those with multiple accounts could not be eliminated. The questionnaire, however, appealed to respondents not to fill the questionnaire more than once and the platform prevented respondents from submitting more than one response from the same account. Lastly, although the sample size was adequate to detect statistical differences, some CIs were stretched, suggesting that the study may benefit from a much bigger sample. Despite these limitations, this is the first study to provide evidence of the CQ controversy during the pandemic whilst controlling the potential confounders during the analysis. Another advantage of our survey is that it was collected when the restrictions were the strictest in the concerned countries. The data collection method was the same across the countries, and people answered on a voluntary basis. Beyond the reduced cost, another key advantage of online surveys is that the questionnaire is available to a great number of people, at any time of the day; also, the data can be processed in real-time.

## Conclusion

In summary, the world faces imperatives to combat dangerous misinformation around COVID-19. In the absence of a known effective therapy, the possibility of a second wave of COVID-19 or another potential public-health emergency, this first population-based survey provided evidence of an avoidable danger of CQ abuse and its associated complications, particularly amongst East Africans. The gross inadequate knowledge and increasing worry shown by Africans in this study suggest the need for regional educational intervention to create awareness and sensitise the public on COVID-19 transmission as well as re-orientate the communities on the dangers of indiscriminate use of CQ during the pandemic. Pharmaco-medical control should be imposed on the acquisition of CQ by governments to control abuse. Public health officers and clinicians have roles to play in discouraging this attitude by highlighting the non-proven use of CQ in treating COVID-19. There is a risk that Africans who resort to CQ might not follow up on legitimate COVID-19 symptoms with their doctors, which in turn, could facilitate the spread of the virus and put their health, and potentially that of others, at risk.
